# Chemical Changes Associated with Increased Acid Resistance of Er:YAG Laser Irradiated Enamel

**DOI:** 10.1155/2014/501357

**Published:** 2014-01-27

**Authors:** Jennifer Manuela Díaz-Monroy, Rosalía Contreras-Bulnes, Oscar Fernando Olea-Mejía, María Magdalena García-Fabila, Laura Emma Rodríguez-Vilchis, Ignacio Sánchez-Flores, Claudia Centeno-Pedraza

**Affiliations:** ^1^Facultad de Odontología, Centro de Investigación y Estudios Avanzados en Odontología (CIEAO), Universidad Autónoma del Estado de México, Jesús Carranza Esq. Paseo Tollocan, Col. Universidad, 50130 Toluca, MEX, Mexico; ^2^Centro Conjunto de Investigación en Química Sustentable (CCIQS), Universidad Autónoma del Estado de México-Universidad Nacional Autónoma de México, Km 14.5 Carretera Toluca-Ixtlahuaca, San Cayetano de Morelos, 50200 Toluca, MEX, Mexico; ^3^Laboratorio de Instrumental, Facultad de Química, Universidad Autónoma del Estado de México, Paseo Tollocan Esq. Paseo Colón, 50120 Toluca, MEX, Mexico

## Abstract

*Background*. An increase in the acid resistance of dental enamel, as well as morphological and structural changes produced by Er:YAG laser irradiation, has been reported. *Purpose*. To evaluate the chemical changes associated with acid resistance of enamel treated with Er:YAG laser. *Methods*. Forty-eight enamel samples were divided into 4 groups (*n* = 12). Group I (control); Groups II, III, and IV were irradiated with Er:YAG at 100 mJ (12.7 J/cm^2^), 200 mJ (25.5 J/cm^2^), and 300 mJ (38.2 J/cm^2^), respectively. *Results*. There were significant differences in composition of irradiated groups (with the exception of chlorine) and in the amount of calcium released. *Conclusions*. Chemical changes associated with an increase in acid resistance of enamel treated with Er:YAG laser showed a clear postirradiation pattern characterized by a decrease in C at.% and an increase in O, P, and Ca at.% and no changes in Cl at.%. An increased Ca/P ratio after Er:YAG laser irradiation was associated with the use of higher laser energy densities. Chemical changes produced by acid dissolution showed a similar trend among experimental groups. Stable or increased Ca/P ratio after acid dissolution was observed in the irradiated groups, with reduction of Ca released into the acid solution.

## 1. Introduction

Enamel is the hardest mineralized biological tissue, in the form of crystallites of hydroxyapatite (96% by weight and 90% by volume). The remaining nonmineral component of enamel is comprised of about 3 wt.% water (8 vol.%), as well as 1 wt.% organic material (2 vol.%) [1].

At least 41 elements of the periodic table have been identified in the chemical composition of the human dental enamel [2]; however, the Ca is the most abundant [3].

The surface of enamel is, perhaps, its most clinically significant region because it is here that dental caries is initiated [1]. The use of different types of lasers for caries preventive purpose has been studied ever since Stern and Sognnaes [4] first suggested the use of ruby laser irradiation.

In 1997, the Er:YAG laser was the first dental laser approved to be used for hard tissue ablation by the US Food and Drug Administration (FDA) [5]. It is a versatile, commercially available dental laser used in both hard and soft tissues, with multiple applications [5–8]. Additionally, an early report suggested that enamel adjacent to the ablated area by Er:YAG laser irradiation exhibited an increase in acid resistance [9]. It has been reported that caries preventive effects induced by Er:YAG laser treatment have been shown to depend on the energy density of the laser, the irradiation time, the focal distance, and the irrigation conditions [10–17].

Recently, there has been a growing interest in the chemical changes that occur in enamel after Er:YAG laser irradiation. Several spectroscopic characterization techniques, including dispersive spectroscopy (WDS) [18], X-ray photoelectron spectroscopy (XPS) [19], X-ray spectroscopy (EDX) [20], Fourier-transform Raman and infrared spectroscopy (FT-Raman and FTIR) [21, 22], and energy-dispersive spectroscopy (EDS) [23] have been used to evaluate changes in the inorganic component of tooth enamel, differences in the calcium/phosphorus (Ca/P) ratio, changes in carbonate content, and modifications in the organic material content and weight percentages (wt.%) of several elements in enamel [19–21, 23]. However, further studies are required to clarify the action mechanisms of Er:YAG laser when enamel acid resistance is achieved.

There are no previous reports that evaluate the chemical composition of human dental enamel on the exact same area of the sample, before and after Er:YAG laser irradiation, as well as subsequent to acid dissolution. For this reason, the aim of the present study was to evaluate the chemical changes associated with acid resistance of enamel treated with Er:YAG laser.

## 2. Materials and Methods

### 2.1. Tooth Selection and Sample Preparation

The study protocol was reviewed and approved by the Research and Ethics Committee at the Autonomous University of the State of Mexico. All subjects enrolled in this study signed an informed consent form. Twenty-four caries-free bicuspid teeth were extracted for orthodontics reasons from patients aged 15–17 years, who reported no use of fluoride products, other than fluoride toothpaste. Teeth with caries, restorations, cracks, and defects in the structure or color of the enamel on the buccal surfaces or fluorosis were excluded. The teeth were stored in a 0.2 (w/v) thymol solution at 4°C for one month until the experiment was performed. Enamel fluorescence was evaluated with a DIAGNOdent pen (DIAGNOdent, KaVo, Biberach, Germany), and only teeth with sound enamel (values of 0–13, according to the manufacturer's specifications) were included in the study [24, 25].

**Figure 1:**
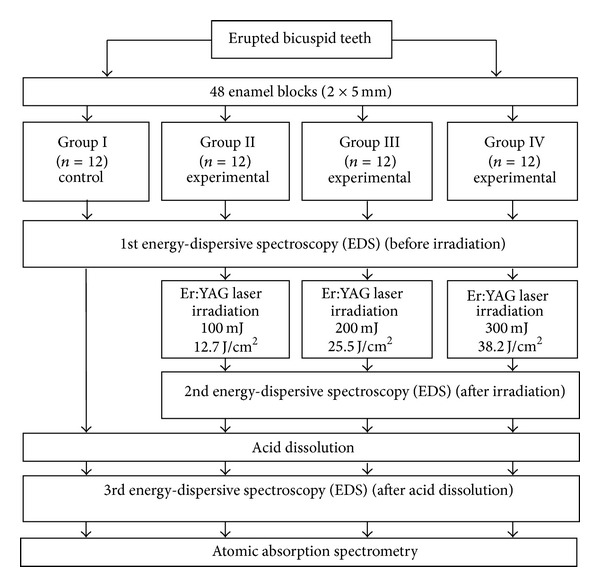
Diagram of the experimental design.

The crown of each tooth was removed using a diamond disc (BesQual, New York, USA) mounted on a low-speed motor (Micromotor M2 Master, M25800011, Drillco Devices Ltd., Miami, FL, USA) under distilled water irrigation to prevent dehydration. The crown was fixed to a glass slide with thermoplasticized epoxy resin (Allied High Tech Products, Rancho Dominguez, CA, USA). Afterwards, a diamond wheel (South Bay Technology, Inc., San Clemente, CA, USA) mounted on a cutter (South Bay Technology, Inc., USA) was used to obtain the samples under constant irrigation. Each tooth was cut to obtain 2 blocks (2 × 5 mm) from buccal surface. A reference point was made on the enamel with a number 1/4 round carbide bur (S.S. White, Lakewood, NJ, USA) and a high-speed handpiece (W&H Dentalwerk, Bürmoos, Austria), with continuous water spray. Finally, the samples were thoroughly washed with distilled water and dried at room temperature prior to EDS evaluation. A total of forty-eight samples were obtained (twelve samples per group) (Figure 1).

### 2.2. Er:YAG Laser Irradiation

An Er:YAG laser system (Lumenis OPUS DUO Er:YAG + CO_2_, Yokneam, Israel) was used to irradiate the samples. We used a fixed wavelength of 2.94 µm, an energy pulse from 100 to 300 mJ (depending on the experimental groups), a pulse repetition rate of 10 Hz, a pulse duration of 250–400 µsec, and an exit sapphire tip with a diameter of 1.0 mm. The forty-eight buccal enamel samples were divided into four groups (*n* = 12): Group I, control (no laser irradiation); Group II, irradiated at 100 mJ (12.7 J/cm^2^); Group III, irradiated at 200 mJ (25.5 J/cm^2^); Group IV, irradiated at 300 mJ (38.2 J/cm^2^). Energy levels were calibrated with the calipers included within the equipment, and the energy delivered was measured periodically with a power meter (LaserMate-P, Coherent Co., Santa Clara, CA, USA).

Irradiation was performed manually from the reference point with a laser spot diameter of 1 mm along the whole sample, in one direction such that the tip was scanned smoothly, perpendicular to the enamel surface of the sample, and without water irrigation. Each sample was irradiated once for 15 s while the tip-sample distance was kept fixed at 1 mm, using the focused laser mode. A sheet of stainless steel (23 mm × 5 mm × 0.5 mm) was fixed to the top of the laser handpiece to ensure the appropriate tip-sample distance. At this tip-sample distance, the exit tip and laser beam had the same diameter, as confirmed by a laminated infrared sensor screen (Lumitek International, Inc., Ijamsville, MD, USA).

### 2.3. Energy-Dispersive Spectroscopy

After each SEM evaluation, the atomic percentages (at.%) of C, O, P, Cl, and Ca were determined by energy dispersive X-ray spectroscopy (Thermo Noran Superdry, USA), in an area of 310 × 210 µm.

### 2.4. Acid Dissolution

Prior to this procedure, each surface of the sample was coated with an acid-resistant varnish, except the experimental area of interest. Subsequently, all samples were individually demineralized in 2 mL of 0.1 M lactic acid, pH 4.8. The samples remained in the demineralization solution for 24 h in an incubator at 37°C and 100% humidity.

### 2.5. Atomic Absorption Spectroscopy

After acid dissolution, samples were rinsed with deionized water into test tube to remove Ca residues from the tooth surface. This procedure was followed in order to obtain the amount of Ca released from the samples by atomic absorption spectrometry (Thermo Elemental Solaar, Marietta, OH, USA).

### 2.6. Statistical Analyses

All data were analyzed using SPSS 19 IBM statistical package (SPSS IBM, New York, NY, USA). The Kolmogorov-Smirnov test was used to assess the data distribution at a significance level of *P* ≤ 0.05. The Friedman and Wilcoxon tests were performed to compare the atomic percentages of the different elements, with a significance level of *P* ≤ 0.05.

The Kruskal-Wallis and Mann-Whitney *U* tests were used to analyze the differences in the Ca concentrations released into solution among the groups.

## 3. Results

### 3.1. EDS Evaluation

The at.% of C, O, P, Cl, and Ca obtained by EDS for all groups are displayed in Table 1. There were statistically significant differences in element composition according to experimental stages in irradiated groups, with the exception of Cl which showed similar values in all groups. The atomic Ca/P ratios showed significant increases in Groups III and IV.

### 3.2. AAS

All experimental groups showed significative lower amounts of Ca released from the enamel upon acid dissolution compared with the control group, which showed the highest amount of released Ca (Table 2).

## 4. Discussion

Over the last decades, many studies have reported that laser can improve the resistance of human dental enamel to acid attacks [9, 11, 15, 17, 26]. In the present study, a lower amount of Ca in the acidic solution of irradiated groups showed that enamel acid resistance was increased under the experimental conditions employed. However, this expected effect was more evident for Groups III and IV.

The chemical changes associated with increased acid resistance of human dental enamel treated with Er:YAG laser were evaluated by EDS analysis. It is worth mentioning that no reports in the current literature have established the concentration of various elements on the same enamel area during consecutive experimental phases (before irradiation, after irradiation, and after acid dissolution). Although the sequential EDS analysis for control group did not show statistically significant differences for at.% after acid dissolution process, this group showed the highest values for released Ca in the acid solution, according to the atomic absorption spectrometry results. Probably, this element was released from enamel subsurface, beyond the sensibility of the EDS analysis.

In contrast to the control group, irradiated groups revealed statistically significant differences for at.% of most of the elements analyzed during the experimental phases. Only Cl did not exhibit a significant change, likely because Cl is found in minimal quantities in the enamel as reported by Losee et al. [2], and minor changes in Cl content are therefore not evident. Consistent with the increased acid resistance shown by AAS, a significant reduction in the at.% of C was observed after laser irradiation, probably resulting from a decrease in carbonate content. Liu and Hsu [21] have suggested the reduction of carbonate and modification of organic matter as mechanisms for dental caries prevention produced by Er:YAG laser irradiation.

Both O and P showed a significant increase in at.% after irradiation, probably by an increase in content of pyrophosphates, associated with heating of tooth enamel [27]. A significant increase in the at.% of Ca was also observed after irradiation, affecting the Ca/P ratio. Contrary to that reported by Mine et al. [19] and de Andrade et al. [20], this ratio did not show a decrease after Er:YAG laser irradiation. However, they did not examine the same enamel area before and after irradiation. In our study, the Ca/P ratio was significantly increased specially when higher energy densities were used, as in the case of Groups III and IV. This ratio remained unchanged when a lower energy density was used (Group II). Nevertheless, additional studies are recommended to evaluate the effect of the energy density on the Ca/P ratio.

During the third phase of EDS analysis (after acid dissolution), a similar trend was shown in the chemical changes produced by the acid dissolution on experimental groups. It was characterized by a decrease in the Ca, P, and O at.%, as well as an increase of C at.% compared with postirradiation values.

The chemical change after acid dissolution showed stable or increased atomic Ca/P ratio among irradiated groups, which showed reduction of Ca released into the acid solution, according to Cecchini et al. [15], who reported that groups irradiated with higher energies than those used in our study, presented an increment in enamel acid resistance, with lower amounts of calcium delivered in the solution in comparison to the control.

## 5. Conclusions

Chemical changes associated with increased acid resistance of enamel treated with Er:YAG laser showed a clear postirradiation pattern characterized by a decrease in C at.% and an increase in O, P, and Ca at.% and no changes in Cl at.%.

An increased Ca/P ratio after Er:YAG laser irradiation was associated with the use of higher laser energy densities.

Chemical changes produced by acid dissolution showed a similar trend among experimental groups, resulting in a decrease of Ca, P, and O at.%, as well as an increase of C at.%.

Stable or increased Ca/P ratio after acid dissolution was shown by irradiated groups, with a reduction of Ca released into the acid solution.

## Figures and Tables

**Table 1:** Mean values and standard deviations of elements' atomic percentages and the Ca/P ratio on the irradiated enamel surface by experimental groups and stages.

Element	Average value and standard deviation										
	Group I: control		Group II: 100 mJ			Group III: 200 mJ			Group IV: 300 mJ		
	Untreated surface	After acid dissolution	Before irradiation	After irradiation	After acid dissolution	Before irradiation	After irradiation	After acid dissolution	Before irradiation	After irradiation	After acid dissolution
Carbon C	8.04^A^ ± 6.36	8.76^A^ ± 4.75	9.31^A^ ± 5.10	5.64^B^ ± 1.87	14.34^C^ ± 7.76	7.66^A^ ± 5.06	0.52^B^ ± 1.80	10.82^A^ ± 3.32	20.81^A^ ± 9.15	0.94^B^ ± 2.24	11.26^C^ ± 6.05
Oxygen O	57.98^A^ ± 5.05	58.09^A^ ± 4.95	62.8^A^ ± 5.82	64.5^B^ ± 4.11	57.13^C^ ± 7.29	66.19^A^ ± 3.94	69.70^B^ ± 1.52	62.98^C^ ± 2.41	53.65^A^ ± 5.32	67.03^B^ ± 4.62	59.35^C^ ± 6.23
Phosphorus P	12.91^A^ ± 1.98	12.69^A^ ± 1.73	11.56^A^ ± 1.25	12.35^B^ ± 1.05	11.93^A^ ± 4.00	11.18^A^ ± 0.75	12.14^B^ ± 0.54	10.65^A^ ± 0.59	10.27^A^ ± 2.17	12.53^B^ ± 1.29	11.02^A^ ± 1.44
Chlorine Cl	0.41^A^ ± 0.08	0.37^A^ ± 0.10	0.31^A^ ± 0.05	0.34^A^ ± 0.05	0.32^A^ ± 0.09	0.32^A^ ± 0.04	0.30^A^ ± 0.15	0.32^A^ ± 0.03	0.27^A^ ± 0.07	0.16^A^ ± 0.17	0.26^A^ ± 0.13
Calcium Ca	20.67^A^ ± 6.19	20.10^A^ ± 6.71	16.02^A^ ± 2.65	17.17^B^ ± 2.39	16.24^A^ ± 5.64	14.65^A^ ± 0.98	17.34^B^ ± 1.11	15.15^A^ ± 1.13	15^A^ ± 4.09	19.34^B^ ± 3.24	18.10^C^ ± 2.79
Ca/P ratio	1.60^A^ ± 0.25	1.58^A^ ± 0.33	1.38^A^ ± 0.10	1.38^A^ ± 0.08	1.36^*A*^ ± 0.10	1.31^A^ ± 0.02	1.43^B^ ± 0.08	1.42^B^ ± 0.07	1.45^A^ ± 0.15	1.54^B^ ± 0.12	1.65^C^ ± 0.20

Groups with different letters are significantly different (*P* ≤ 0.05).

**Table 2:** The mean values and standard deviations of released calcium into acid solution per group.

Group	*N*	Calcium (mg/L) ± SD
I	12	0.92^A^ ± 0.47
II	12	0.27^B^ ± 0.15
III	12	0.14^C^ ± 0.03
IV	12	0.17^C^ ± 0.08

Groups with different letters are significantly different (*P* ≤ 0.05).
